# Effect of macrophages in semen on sperm quality

**DOI:** 10.1186/s12958-021-00724-1

**Published:** 2021-03-04

**Authors:** Gangxin Chen, Beihong Zheng

**Affiliations:** Reproductive Medicine Center, Fujian Provincial Maternity and Children‘s Hospital, Affiliated Hospital of Fujian Medical University, Daoshan Road 18, Fuzhou, Fujian 350001 China

**Keywords:** Macrophages, Semen quality, AsAb, IL-10, IL-12

## Abstract

**Background:**

This was a cross-sectional study in China which analyzed the levels of macrophages (Mφ) in semen and evaluated the influence of Mφ levels in semen on sperm quality.

**Methods:**

The subjects involves 78 males, 25- to 35-year old. The samples were divided into a low group (Mφ < 6 × 10^5^/ml) and a high group (Mφ > 6 × 10^5^/ml). Evaluation included consideration of the influencing factors of male semen quality, macrophage concentration, sperm motility, morphology, membrane integrity DNA fragmentation index (DFI), anti-sperm antibodies (AsAb), IL-10, and IL-12 in semen.

**Results:**

There was no difference in the physical or chemical indices of the semen, sperm concentration, AsAb, IL-10, or IL-12 between the two groups (*P* > 0.05). The percentage of sperm forward motility (PR%), the rate of normal sperm shape, and the integrity of cell membranes in the low group were higher than those in the high group (*P* < 0.05), while the percentage of sperm inactivity (IM%), the rate of sperm head deformity, the rate of deformity in the neck and middle segment, the sperm deformity index (SDI), the teratozoospermia index (TZI), and the sperm DFI in the low group were lower than those in the high group (*P* < 0.05). The concentration of Mφ in the semen was linearly correlated with sperm concentration, sperm PR%, IM%, sperm normal shape rate, head deformity rate, neck and middle deformity rate, SDI, TZI, sperm DFI, and sperm cell membrane integrity (*P* < 0.05), but there was no linear correlation with IL-10 or IL-12 (*P* > 0.05).

**Conclusions:**

The Mφ concentration in semen is not significantly correlated with semen volume or sperm concentration, but negatively correlated with sperm motility, morphology, cell membrane integrity, and DNA damage rate. There is no significant correlation between the macrophages and the concentration of IL-10 or IL-12.

## Background

Over the past 50 years, male fertility has continued to decline. The quality of men’s semen has reduced; among which, the concentration of sperm and the total number of sperm have decreased by more than 50% compared with the past [1, 2]. At present, about 15% of the population of childbearing age suffer from infertility, and almost half of the infertility factors are related to men, of which 34% are unable to find the cause [3]. The general decline in male fertility has become a common problem facing all mankind.

Immune factors are believed to be related to the decline in male fertility. There are a small number of immune cells in male semen (mainly neutrophils, Mφ, and lymphocytes), which contribute to immune defense and immune surveillance of the reproductive system [4]. The cells in semen other than sperm are called round cells [5]. In these round cells, if the concentration of leukocytes exceeds 1 × 10^6^/ml, sperm quality may decrease. Although the effect of monocytes or Mφ in semen on sperm quality is not yet clear [5], it is widely accepted that Mφ has the function of antigen presentation, mainly by secreting a variety of cytokines, enzymes, reactive oxygen species (ROS) and other substances, together with other immune cells to maintain the immune balance of the testis [6]. In this study, we analyzed the relationship between Mφ in semen and sperm quality parameters, so as to provide a reference basis for the diagnosis and treatment of male diseases. In addition, some patients experience unexplained repeated pregnancy failure. The results of this study are expected to provide new ideas for the diagnosis and treatment of patients who experience unexplained repeated pregnancy failure in the field of assisted reproduction.

## Materials and methods

### Subjects

Semen samples from 78 men (aged 20 to 35 years old) were collected from the Reproductive Center of Fujian Women’s and Children’s Hospital between 2018 and 2019. During their physical examination, genital erection and sperm excretion are normal, and no organic lesions were found in the reproductive organs such as testis, epididymis, seminal vesicles and prostate. The participants did not have varicocele or endocrine diseases, and each participant had a normal karyotype and reproductive history. The participants were negative for Mycoplasma, Chlamydia, Ureaplasmosis, Trichomonas and Gonorrhea. In addition, other related factors affecting semen quality (high BMI, orchitis, epididymal orchitis, smoking, drinking, drug use and poor hygiene) were excluded.

The study was approved by the Ethics Committee of Fujian Women’s and Children’s Hospital, with the approval number of 2019KY105. All participants provided informed consent in accordance with the regulations of the Ethics Committee. All methods are carried out in accordance with relevant guidelines and national regulations.

### Sample collection

Before semen extraction, the patients were required to abstain from sex for 2 to 7 days, followed by masturbation to obtain semen. Semen samples were collected in sterile sperm collection cups and immediately placed in a constant temperature incubator at 37 °C for liquefaction. We recorded the color, volume, liquefaction time, pH, viscosity and other physical and chemical parameters of semen. In addition, the birth history and physical examination results of each patient were also recorded.

### Analysis of macrophages

The samples were Pap stained according to WHO guide 5, and standard macrophages (Institute of Cell Research, Chinese Academy of Sciences) were used to observe the morphology of Mφ in semen. The concentration of macrophages was detected by flow cytometry (BDCantoII, USA) and CD14 (Biolegend, USA). Three specific test tubes were used: Test tube 1 contained upstream sperm as a blank control. Tube 2 has a standard macrophage cell line as a positive control. The test tube 3 contains a specimen. According to the operating instructions, the CD14 antibody fluorescein isothiocyanate (CD14-FITC) was added in sequence and placed in a dark room at an appropriate temperature for 20 min. 1000 μl PBS was then added and gently mixed; washed twice at 1500 r/min, and centrifuged for 5 min before being tested on the machine. Next, we use the software to draw a dot plot of all collected cells through CD14-FITC, and use the FITC positive area as gate P1. The samples are divided into low concentration group (Mφ < 6 × 105/ml) and high concentration group (Mφ > 6 × 105/ml) [7].

### Sperm kinetic analysis

According to the standards of the WHO guidelines [5], the Makler counting chamber (Haifa, Israel) is used for sperm kinetic analysis, including sperm concentration, progressive movement (PR%), non-progressive movement (NP%) and motility (IM%) and other parameters, which were then compared and verified with the analysis results of computer automatic semen analyzer.

### Sperm morphology analysis

**Fig. 1:**
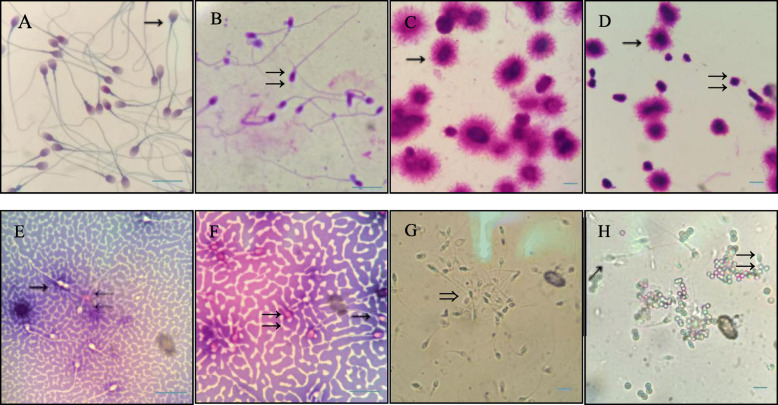
The sperm quality effect chart. Sperm morphology (Pap staining). **a** Normal sperm morphology. **b** Abnormal sperm morphology. Sperm DNA fragments (SCD). **c** Sperm with normal DNA spread. **d** Sperm with abnormal DNA spread sperm membrane integrity (aniline staining). **e** Sperm with intact cell membranes. **f** Sperm with incomplete cell membranes. Anti-sperm antibody detection (MAR). **(G)** Partial sperm agglutination. **h** Sperm being braked (→ indicates normal state, means abnormal state). Magnification: **a**, **b**, **e**, **f** × 1000; **c**, **d**, **g**, **h** × 400). Bar = 50 μm

According to the standards of the WHO guidelines [5], the samples were stained by Pap staining (Anhui Anke Biotechnology Co, Ltd., Anhui, China), and at least 200 sperm were observed in each sample. Normal sperm shape rate, head deformity rate, neck and middle segment deformity rate, major segment deformity rate, cytoplasmic residual droplet rate, the sperm malformation index (SDI), the abnormal sperm index (TZI) were evaluated according to the WHO criteria [5]. See Fig. 1.

### Sperm DFI analysis

The samples were stained using sperm chromatin diffusion (SCD) staining method described in the literature [8, 9]. According to the operation manual (Anhui Anke Biotechnology Co., Ltd., Anhui, China), the semen is fixed, acid denatured, lysed, diffused, dehydrated and dyed. The large halo on the sperm head indicates the intact sperm DNA, while no halo or only a small halo indicates that the sperm DNA is incomplete. Randomly at least 500 sperm in each sample were observed to calculate the DNA fragmentation index (DFI) of the sperm (DFI = number of sperm with positive DNA fragmentation rate/total number of sperm observed × 100%). See Fig. 1.

### Sperm cell membrane integrity analysis

The samples were subjected to Eosin-aniline black staining^5^, in which the staining solution (BRED Life Science, Shenzhen, China) was mixed gently with semen for 30 s. At least 200 sperm were observed under the microscope to evaluate the sperm survival rate. A black or dark red sperm head indicates abnormal sperm, while colorless or light red indicate normal sperm (sperm survival rate = number of sperm that do not damage the cell membrane / total number of sperm observed × 100%). See Fig. 1.

### Anti-sperm antibody analysis

The mixed anti-globulin reaction (MAR) reagent from BRED Life Science (Shenzhen, China) is used to detect anti-sperm antibodies on the sperm surface. Magnetic beads attached to the surfaces of the sperm represented a positive sperm antibody. When the number of immobilized sperm accounted for more than 50% of the total sperm, semen AsAb was positive [5]. as shown in Fig. 1**.**

### Cytokines analysis

According to the instructions of the ELISA kit (American Standard Biotechnology Co, Ltd., Jiangsu, China), the semen was centrifuged at 5000 g for 30 min with the seminal plasma removed; followed by addition of antibodies IL-10, IL-12. Finally color developing solution and stop solution were added; the concentration of IL-10, 12 in the semen is detected at a wavelength of 450 nm.

### Statistical analysis

Statistical analysis was performed using SPSS17.0 (IBM, Armonk, NY, USA). The measurement data were expressed as mean ± standard deviation, and the independent sample t-test was used determine the significant difference of means between two groups; and the counting data were expressed by rate (%) and compared by a χ2 test. The coefficient of determination R^2^ was calculated to indicate the linearity between macrophage concentration and sperm quality, drawn by GraphPad Prism 6.07(San Diego, CA, USA). *P*-value < 0.05 indicates a significant difference.

## Results

### Analysis of macrophages

**Fig. 2:**
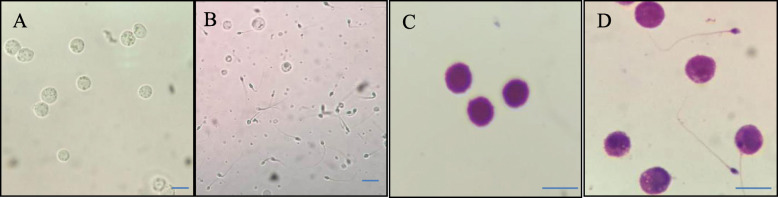
Morphological comparison of macrophages in semen with standard macrophages. **a** Morphology of standard macrophages. **b** Morphology of macrophages in semen. **c** Morphology of standard macrophages after Pap staining. **d** Morphology of macrophages in semen after Pap staining (Magnification: **a**, **b** × 400; **c**, **d** × 1000). Bar = 50 μm

**Fig. 3:**
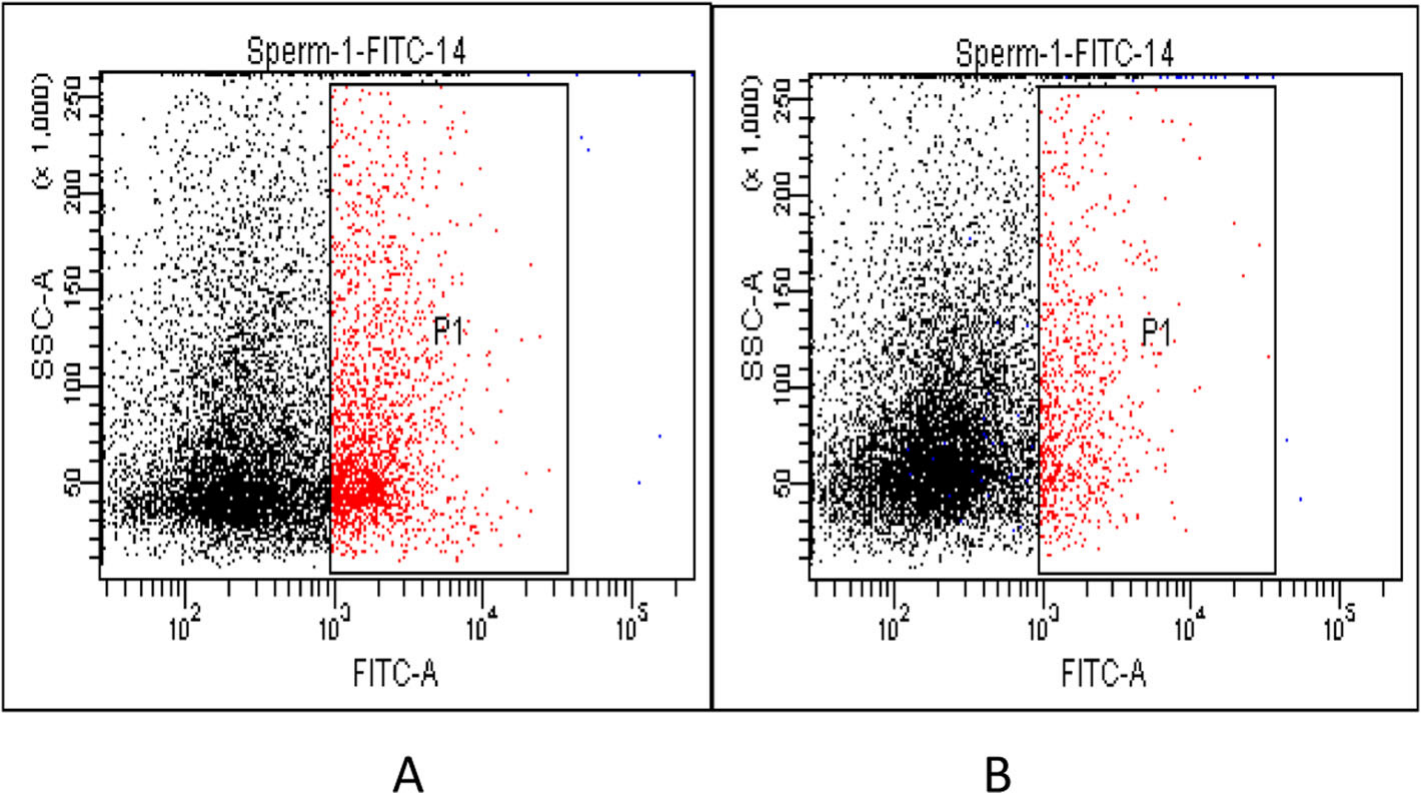
Detection of macrophages in semen after flow cytometry. **a** Positive group of macrophages in semen (CD14+). **b** Negative group of macrophages in semen (CD14-)

Some round cells, similar to the standard macrophages, could be seen in the semen (Fig. 2). Pap smear staining showed that these cells had less cytoplasm, with the semi-elliptical nucleus and more than three-quarters of nucleus-to-whole cell volume ratio (Fig. 2). In all 78 semen cases, of which 34 were with macrophages < 6 × 10^5^/ml (mean 2.17 ± 1.76) and 44 cases with macrophages > 6 × 10^5^/ml (9.45 ± 2.47) (Fig. 3).

### General situation analysis

There was no significant difference in age, abstinence time, or primary infertility rate between the two groups (*P* > 0.05). Semen liquefaction time of both groups was less than 30 min. There was no wire drawing phenomenon in the liquefied semen, and there was no significant difference in semen volume, pH, or any other physical or chemical parameters **(**Table 1).

**Table 1:** Comparison of condition and physical and chemical parameters of semen

Related parameters	Low group (*n* = 34)	High group (*n* = 44)	t/X^**2**^	***P***-value
Mφ (10^5^/ml)	2.17 ± 1.76	9.45 ± 2.47	−15.17	0.00
Age (years)	30.29 ± 4.88	30.57 ± 3.68	−0.28	0.77
BMI	19.67 ± 1.04	19.95 ± 1.14	−1.10	0.27
Primary infertility rate (%)	79.4 (27/34)	79.5 (35/44)	0.00	0.98^a^
Abstinence time (day)	3.85 ± 0.99	4.27 ± 1.26	−1.59	0.11
Semen volume (ml)	3.24 ± 0.91	3.52 ± 1.16	−1.14	0.25
pH	7.46 ± 0.19	7.40 ± 0.42	1.34	0.18

^a^X^2^ Test

### Sperm kinetic analysis

**Fig. 4:**
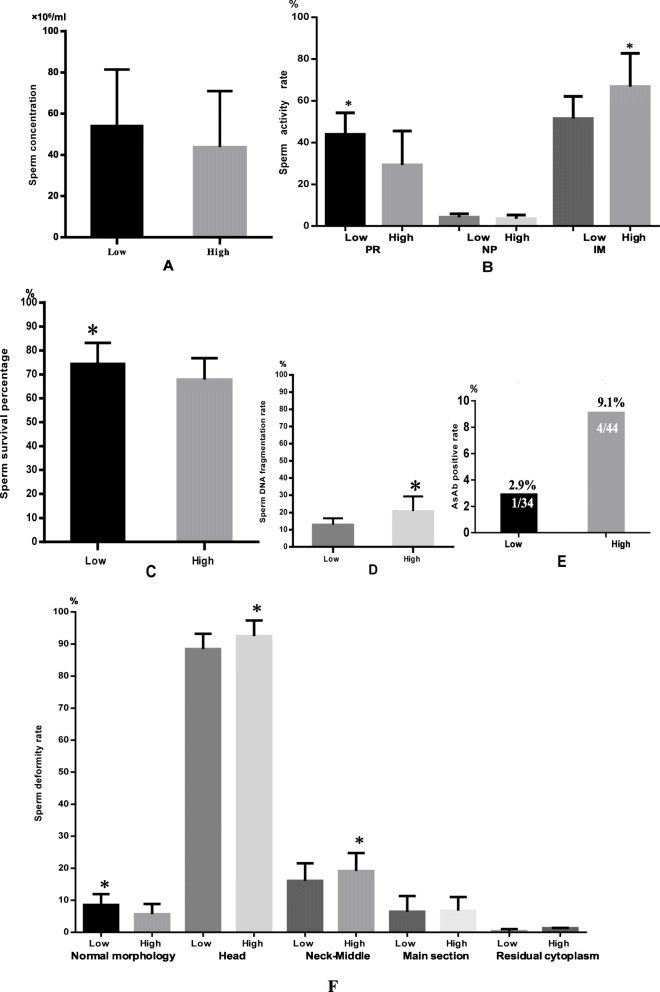
Comparison of sperm quality parameters between the two groups. **a** Difference in sperm concentration (**b**) Comparison of sperm motility (**c**) Comparison of sperm membrane integrity (**d**) Comparison of sperm DFI (**e**) Comparison of AsAb (**f**) Comparison of sperm morphology. *Indicates statistical difference between the two groups (*P* < 0.05)

The sperm concentration and NP% in the low concentration group were slightly higher than those of the high concentration group, but there was no significant difference between the two (*P* > 0.05). The PR% of sperm in the low concentration group was higher than that of the high concentration group, and the IM% of sperm in the low concentration group was lower than that of the high concentration group (*P* < 0.05) [Table 2, Fig. 4].

**Table 2:** Comparison of sperm quality parameters between the two groups

Sperm quality parameters	Low group (*n* = 34)	High group (*n* = 44)	t/X^**2**^	***P***-value
Sperm concentration (10^6^/ml)	54.14 ± 27.23	44.45 ± 26.55	1.58	0.11
PR%	43.94 ± 10.30	29.29 ± 16.22	4.85	0.00
NP%	4.29 ± 1.62	3.56 ± 1.73	1.88	0.06
IM%	51.50 ± 10.63	66.84 ± 15.92	−4.84	0.00
Normal morphology rate (%)	8.52 ± 3.37	5.68 ± 3.14	3.83	0.00
Head deformity rate (%)	88.44 ± 4.76	92.52 ± 4.82	−3.72	0.00
Neck deformity rate (%)	16.05 ± 5.49	19.13 ± 5.63	−2.41	0.01
Main segment deformity rate (%)	6.44 ± 4.87	6.75 ± 4.26	−0.29	0.76
Slurry drop rate (%)	1.38 ± 0.65	1.45 ± 0.97	−0.39	0.69
SDI	1.10 ± 0.11	1.19 ± 0.13	−3.28	0.00
TZI	1.20 ± 0.10	1.26 ± 0.12	−2.44	0.01
DFI (%)	12.97 ± 3.69	20.86 ± 8.39	−5.10	0.00
Sperm membrane integrity (%)	74.52 ± 8.61	67.86 ± 8.88	3.32	0.00
AsAb positive rate (%)	2.9 (1/34)	9.1 (4/44)	0.40	0.52^a^
IL-10 (pg/ml)	464.17 ± 60.53	465.63 ± 57.21	−0.10	0.91
IL-12 (pg/ml)	81.41 ± 13.09	80.87 ± 10.90	0.19	1.84

^a^X^2^ Test

### Sperm morphology analysis

Sperm stained with Pap clearly shows the morphological features of each part, and there is a significant difference between the normal and abnormal sperm (Fig. 2). The normal morphology rate of sperm in the low concentration group is higher than that in the high concentration group. The incidence of head deformity, neck and middle segment deformity, SDI and TZI in the low concentration group was lower than that in the high concentration group (*P* < 0.05). There was no significant difference in the rate of major segmental deformities or cytoplasmic residual droplets between the two groups (*P* > 0.05 [Table 2, Fig. 4]).

### Sperm DFI analysis

After SCD staining, the sperm head forms a halo of different thickness, where no halo or only a small halo around the sperm indicates abnormal sperm DNA integrity (Fig. 1). Our results showed that the sperm DFI of the low-concentration group was significantly lower than that of the high-concentration group (*P* > 0.05 [Table 2, Fig. 4]).

### Sperm cell membrane integrity analysis

The integrity of cell membranes could be clearly identified due to the eosin black staining of spermatozoa (Fig. 1). The results showed that the sperm survival rate of the low concentration group was higher than that of the high concentration group (*P* < 0.05 [Table 2, Fig. 4]).

### Anti-sperm antibody analysis

Partial sperm agglutination could be seen in the semen with positive anti-sperm antibodies. The antibody-positive sperm are attached to the magnetic beads with an obvious immobilization effect (Fig. 1). The results showed that the positive rate of anti-sperm antibodies in the low-concentration group was lower than that in the high concentration group, but there was no significant difference between the two (*P* > 0.05 [Table 2, Fig. 4]).

### Cytokines analysis

The concentration of IL-10 in semen was significantly higher than that of IL-12. There was no significant difference in the concentration of IL10 and IL12 between the two groups. There was no significant correlation between the concentration of macrophages and the concentration of IL-10 and IL-12 in semen (Table 2, Fig. 4).

### Macrophages and sperm quality parameters analysis

**Fig. 5:**
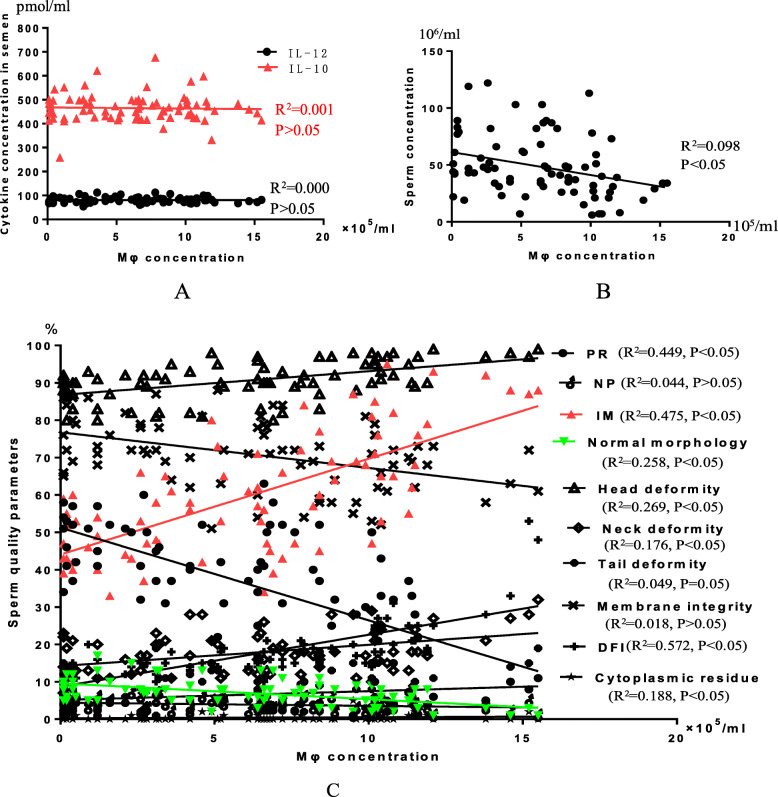
The linear relationship between macrophages and sperm quality. **a** The relationship between macrophages and cytokine. **b** The relationship between macrophages and sperm concentration. **c** The relationship between macrophages and sperm quality parameters. R^2^ represents the degree of correlation; *P* < 0.05 indicates there is a linear relationship between them

There was a significant correlation between macrophages in semen and most sperm kinetic parameters, sperm morphology, DFI, and the survival rate (*P* < 0.05), but there was no significant correlation between macrophages and the positive rate of anti-sperm antibodies (*P* > 0.05 [Fig. 5]).

## Discussion

Macrophages originate from the interstitial tissue of the testis and epididymis, and account for 25% of the total number of testicular stromal cells [10]. A small number of macrophages in semen play an important role in maintaining sperm quality [7]. When an infection occurs in the male reproductive system or the “blood-testis” barrier is destroyed, the macrophages count in the semen will increase significantly [4, 10]. The phenomenon of macrophages engulfing sperm in semen may be a signal that the immune response of the reproductive system is activated [10]. The increase of macrophages in semen leads to a decrease in sperm count, concentration, and vitality [10–13]. Some studies showed that the increase of the number of macrophages in semen does not reduce the quality of the sperm; however, there are studies showing that macrophages in semen are beneficial for improving male fertility [14, 15]. This difference may be due to various identification methods of white blood cell subpopulations in semen and different evaluation criteria of sperm quality. It may also be caused by the different ratio of monocytes and macrophages in semen. When the human body is in a state of immunosuppression, the function of monocytes dominates. After activating the immune function, monocytes will rapidly transform into macrophages [16, 17].

Under physiological conditions, macrophages are in an immunosuppressive state. Once activated by related stimulators, they can differentiate into different subtypes [18, 19]. The mononuclear macrophage cells possess a few labeled antibodies [20–23]. In this study, CD14 was used as a marker antibody to detect macrophages in semen, because none of the semen cells express CD14 except for monocytes-macrophages. The study found that the concentration of macrophages in semen ranged from 0 to 15.5 × 105/ml, and the concentration of macrophages in the high group was nearly 5 times that of the low group (*P* < 0.05). The ratio of macrophages is 0 × 105/ml to 5.3 × 105/ml (mean 2.17 × 105/ml ± 1.67 × 105/ml), and the ratio of macrophages in the high group is 6.1 × 105/ml 15.5 × 105 /ml (mean 9.45 × 105/ml ± 2.47 × 105/ml), with consistent results to other related work [11].

There was no significant difference in sperm concentration, semen volume or other physical and chemical parameters between the two groups, but significant difference in the kinetic parameters (PR%, IM%) between the two, which is consistent with the conclusion from Kuzelova et al. [12]. Semen is composed of sperm and seminal plasma, in which seminal plasma (mainly prostate fluid and seminal vesicle fluid) accounts for more than 90% of semen volume, while sperm accounts for only 5% of semen volume. The functions of the prostate and seminal vesicles play a vital role in sperm concentration and semen volume. Sperm is produced in the testis and stored in the epididymis. Sperm can only obtain energy under the action of modifiers in seminal plasma to promote mitochondrial ATP, production and sperm movement; The above series of processes require the participation of macrophages; therefore, there is a certain correlation between sperm kinetic parameters and macrophages.

The content of macrophages in semen is closely related to sperm morphology, DFI and the integrity of sperm cell membranes. The incidence of normal sperm morphology in the high-concentration group was lower than that in the low-concentration group, while the rates of sperm head deformity, neck and mid-end deformity, SDI and TZI were higher than those in the low-concentration group. There is a significantly positive correlation between the number of macrophages in sperm and sperm DFI, while a significantly negative correlation between macrophages and sperm membrane integrity, which is consistent with the results of related reports [11, 24, 25]. The macrophages in semen come from the testicular interstitium, which is an essential auxiliary cell in the process of spermatogenesis. They interact with supporting cells such as testicular stromal cells and testicular stromal cells to maintain the microenvironmental stability of the testicular stromal cells [26, 27]. Macrophages are mainly immunomodulated by antigen presentation, interleukin, nitric oxide, tumor necrosis factor, 25-hydroxycholesterol, etc. [26].

When a foreign antigen appears in sperm, immune cells will produce immunoglobulins (anti-sperm antibodies) that can specifically recognize and bind to the corresponding antigen. The results of this study showed that in lower concentrations of macrophages, there are fewer AsAb positive samples (2.9% vs. 9.1%), with no statistical difference (*P* > 0.05). This observation is inconsistent with other related studies [7] due to the fact that we excluded high-risk AsAb populations in the initial sample screening.

Cytokines are mainly secreted by activated immune cells and can bind to target cells to mediate immunity and inflammation. IL-12 secreted by macrophages is mainly involved in immune monitoring, and IL-10 secreted is mainly involved in immune down-regulation [28, 29]. The results showed that there was no significant correlation between the content of macrophages and the levels of IL-10 and IL-12 in the two groups, which means that the direction of macrophage immunity is independent of the number of cells. The concentration of IL-10 in the semen of both groups was higher than that of IL-12, which means that regardless of the concentration of macrophages in the semen, its main role is to mediate the inflammatory response of the immune system. The immunosuppressive effect of macrophages on semen is significantly larger than that of immune monitoring.

It is of great significance to investigate the mechanism of monocytes and macrophages in semen that affect sperm quality. For example, (1) the decrease of macrophages in the testis will result in the obstruction of sperm formation or maturation in the seminiferous tubules of the testis [30]. (2) Cytokines produced by macrophages in semen can activate other immune cells. It cooperates with these immune cells and ultimately affects the quality of sperm [26]. (3) Macrophages in semen can secrete ROS, and excessive ROS cause adverse effects on sperm capacitation, acrosome reaction, sperm kinetics, morphology and sperm DNA [4, 31–33]. (4) Macrophages can indirectly mediate the function of neutrophils. The increase of neutrophils in semen has a certain correlation with the occurrence of male teratogenicity [34, 35]. In response to the above-mentioned problem of increased macrophages, the current treatment of high-dose macrophages for men mainly includes antibacterial therapy, antioxidant therapy and immunotherapy. According to the above reasons for the increase of macrophages, the current treatments for high levels of macrophages in men mainly include antibacterial therapy, antioxidant therapy and immunotherapy [36, 37].

In summary, there are certain correlations between the macrophages and sperm kinetics, morphology, sperm cell membrane integrity, sperm DFI, SDI, and TZI. The results of our study indicate that macrophages in semen are critical to sperm quality. Macrophages at low concentrations can protect sperm from foreign antigens, but macrophages with a high concentration impair sperm quality.

Macrophages play an important role in ensuring sperm quality. The examination of the relationship between macrophages in semen samples and multiple sperm quality parameters provides reference values for the diagnosis and treatment of male diseases. In addition, in the field of assisted reproduction technology (ART), some patients suffer repeated pregnancy failures, but fail to find the exact causes. This study is expected to provide new diagnosis and treatment ideas for these patients, given the macrophages in semen having a certain correlation with sperm motility and morphology, men with poor sperm motility and morphology are encouraged to monitor the level of macrophages.

## Data Availability

The datasets used and/or analysed during the current study are available from the corresponding author on reasonable request.

## Abbreviations

Mφ: Macrophages
DFI: DNA fragmentation index
AsAb: Anti-sperm antibodies
IM: Immotility
NP: Non-progressive motility
PR: Progressive motility
SDI: Sperm deformity index
TZI: Teratozoospermia index
WHO: World Health Organization
ROS: Reactive oxygen species
